# Saint Paul, the Apostle, and the Gastaut-Geschwind syndrome

**DOI:** 10.1590/1980-5764-DN-2024-0165

**Published:** 2024-12-16

**Authors:** Leonardo Cruz de Souza, Guilherme Nogueira Mendes de Oliveira, Leandro Boson Gambogi, Ana Paula Gonçalves, Antônio Lúcio Teixeira

**Affiliations:** 1 Universidade Federal de Minas Gerais, Faculdade de Medicina, Departamento de Clínica Médica, Belo Horizonte MG, Brazil.; 2 Universidade Federal de Minas Gerais, Instituto de Ciências Biológicas, Programa de Pós-Graduação em Neurociências, Belo Horizonte MG, Brazil.; 3 Universidade Federal de Minas Gerais, Grupo de Neurologia Cognitiva e do Comportamento, Belo Horizonte MG, Brazil.; 4 Universidade Federal dos Vales do Jequitinhonha e Mucuri, Faculdade de Medicina, Diamantina MG, Brazil.; 5 Hospital Felício Rocho, Núcleo Avançado de Tratamento das Epilepsias, Belo Horizonte MG, Brazil.; 6 Faculdade Santa Casa Belo Horizonte, Belo Horizonte MG, Brazil.; 7 University of Texas Health Science Center at Houston, McGovern Medical School, Department of Psychiatry and Behavioral Sciences, Neuropsychiatry Program, Texas, United States of America.

**Keywords:** Epilepsy, Personality, Behavior, Epilepsia, Personalidade, Comportamento

## Abstract

The interface between epilepsy and religiosity has been a long-standing matter of debate. Epilepsy has affected several religious leaders throughout history. Hyperreligiosity may be observed in patients with temporal lobe epilepsy as a component of the so-called Gastaut-Geschwind syndrome which involves other behavioral and personality traits such as hyposexuality, viscosity, philosophical concerns, sense of personal destiny, hypergraphy, emotionality, and irritability. Saint Paul, the Apostle, probably had temporal lobe epilepsy. He was a genius man of intellectual excellence and refined culture, whose life and writings exerted a decisive influence on Western history. The current paper investigates the elements of Gastaut-Geschwind syndrome in Saint Paul’s life and Epistles and discusses the potential influence of these traits on Pauline theology.

## RESUMO

A interface entre epilepsia e religiosidade é debatida desde a Antiguidade. A epilepsia acometeu vários líderes religiosos ao longo da História. A hiper-religiosidade pode ser observada em pacientes com epilepsia do lobo temporal como um componente da chamada síndrome de Gastaut-Geschwind, que envolve outros traços comportamentais e de personalidade, como hipossexualidade, viscosidade, preocupações filosóficas, senso de destino pessoal, hipergrafia, emotividade e irritabilidade. São Paulo, o Apóstolo, provavelmente tinha epilepsia do lobo temporal. Ele foi um homem de excelência intelectual e cultura refinada, cuja vida e escritos exerceram influência decisiva na história ocidental. O presente artigo investiga os elementos da síndrome de Gastaut-Geschwind na vida e nas Epístolas de São Paulo e discute a potencial influência desses traços na teologia paulina.

## INTRODUCTION

The reciprocal relations among epilepsy, behavior, and religiosity have attracted the attention of philosophers, scientists, and physicians across centuries. Babylonian texts, for example, report exacerbated religiosity in epileptic patients^1^. In recent times, hyperreligiosity has been observed in people with temporal lobe epilepsy (TLE)^2,3,4^.

Hyperreligiosity is a component of the so-called Gastaut-Geschwind syndrome (GGS) which comprises behavioral and personality traits observed in some patients with TLE^2,5^. The GGS is composed of hyposexuality, viscosity, a sense of personal destiny, hypergraphy, emotionality, irritability, and hyperreligiosity. While the full syndrome seems to be rare among people with TLE^6^, isolated traits such as hyperreligiosity are frequently noted, and TLE patients with hyperreligiosity have more GGS elements than non-religious TLE patients^2^.

Another branch of investigation about the relations between epilepsy and religiosity is provided by biographical accounts of historical religious leaders who had the “falling sickness”. Epilepsy has affected several religious figures such as the prophet Ezekiel, Buddha, and Joseph Smith^6^. Saint Paul, the Apostle, was also a prominent religious leader who probably had epilepsy^5,6,7,8,9^.

While previous studies addressed the possible diagnosis of epilepsy of Saint Paul^7,8^, not much attention has been given to his personality. The present paper explores elements of GGS in Saint Paul’s life and Epistles and discusses the potential influence of these traits on his theological tenets.

### Saint Paul’s life

Saint Paul, the Apostle, was born in Tarsus (modern territory of Turkey) in 15 AD and died in Rome (c. 62–64 CE)^10^. He was a Greek-speaking Jew whose original name was Saul. Until his conversion, Saul was a Pharisee and a tenacious opponent of early Christians. Indeed, during half of his life, Saul was a persecutor of Christians, dragging and throwing them into prison (Acts 8:3), and approving their execution (Acts 8:1). However, according to the Bible, an event changed him. On his way to Damascus (modern Syria), *“suddenly there shined round about him a light from heaven”* (Acts 9:3) and he heard a voice, which is attributed to Jesus (Acts 9:5). Based on the biblical report, Saul fell on the ground, and started trembling (Acts 9:6). After arising from the ground, Saul was blind (Acts 9:8) *“and he was three days without sight, and neither did eat nor drink”* (Acts 9:9).

**Figure 1:**
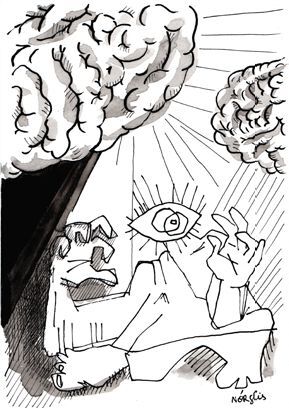
Brainstorm.

Upon undergoing this experience (Figure 1), Saul changed his name to Paul and engaged in missionary trips, spreading the Christian Gospel and founding churches in Asia Minor and Mediterranean Europe. He wrote letters to Christian communities across the modern territories of Syria, Turkey, Greece, and Italy. These texts became canonical and constitute approximately half of the New Testament, markedly influencing the history of Christianity.

Some authors^5,6,7,8^ argue that his conversion to Christ on the road to Damascus might have been an epileptic seizure followed by a prolonged post-ictal blindness. Some arguments corroborate that Saint Paul suffered from TLE. In support to this hypothesis, he complained of a *“thorn in the flesh”*, a chronic infirmity (2 Corinthians 12:7; Galatians 4:13–14) manifesting with recurrent attacks of physical suffering^5,7,8^; he also experienced ecstatic and depersonalization states (2 Corinthians 12:1–4), which would be epileptic auras.

### Elements of Gastaut-Geschwind syndrome on Saint Paul’s life and oeuvre

#### Hyperreligiosity

Religion was a matter of central interest to Saul/Paul throughout his life. Before his conversion, he dedicated himself to persecuting Jews who accepted Jesus’ word. After his Revelation, Paul became a fervent preacher of the Gospel to the Gentiles, devoting himself to missionary trips and pastoral correspondences. Due to his missionary activity as a Christian preacher, Saint Paul was arrested and tortured (Acts 16:19–24; 2 Corinthians 11:24,25). According to the Christian tradition, Saint Paul was executed by Emperor Nero in Rome (64 CE). The centrality of faith and religious practice can also be inferred from the following excerpts (Table 1):

*“For to me to live is Christ, and to die is gain.”* (Philippians 1:21).
*“For of him, and through him, and to him, are all things: to whom be glory for ever. Amen”.* (Romans 11:36).
*“Whether therefore ye eat, or drink, or whatsoever ye do, do all to the glory of God.”* (1 Corinthians 10:31).

**Table 1:** Elements of Gastaut-Geschwind syndrome in Saint Paul’s texts.

Personality Trait	Source
Hyperreligiosity	Acts 17:28; Romans 11:36; 1 Corinthians 10:31; Colossians 1:17,18; Philippians 1:21
Sense of destiny	Romans 1:1; 1 Corinthians 1:1; 2 Corinthians 1:1; Galatians 1:1; 11,12; 1 Timothy 1:16; 2 Timothy 1:1; 11,12; Philippians 3:17
Philosophical concerns	Acts 11:18-34; Romans 8:28–39; 1 Corinthians 12:12–30; 1 Corinthians 13; Ephesians 1:3–14; 1 Thessalonians 4:13–18
Hypermoralism	Romans 1:27–31; Galatians 5:19–21; Ephesians 5:3–5; Colossians 3:5,6
Hyposexuality	1 Corinthians 7:1,2; 7–9
Hypergraphy and viscosity (in thinking)	Romans 1:28–31; Romans 8:38,39; 2 Corinthians 6:4–10; 2 Corinthians 11:24–27; 2 Corinthians 12:20; Galatians 5:19–23
Irritability and Emotionality	Acts 15:2; Acts 15:36–40; Galatians 2:11–14; Romans 8:31–39; 1 Corinthians 1:27,28; 2 Corinthians 11:1–30; Philippians 3:2

### Sense of personal destiny

When addressing himself to his readers, Saint Paul emphasized that God committed himself to preaching the Gospel (Table 1). Moreover, he was convinced that his message was directly transmitted to him by God: *“But I certify you, brethren, that the gospel which was preached of me is not after man. For I neither received it of man, neither was I taught it, but by the revelation of Jesus Christ.”* (Galatians 1:11–12). He also had a marked sense of mission, believing he had a special role in God’s Plan of Salvation: *“Whereunto I am appointed a preacher, and an apostle, and a teacher of the Gentiles. For the which cause I also suffer these things: nevertheless, I am not ashamed: for I know whom I have believed, and am persuaded that he is able to keep that which I have committed unto him against that day.”* (2 Timothy 1:11–12)*.*

This firm belief is also evident in other biblical excerpts (Table 1); for example, when he wrote to Corinthians about communion and the Lord’s supper (*“For I have received of the Lord that which also I delivered unto you”;* 1 Corinthians 11:23) and in the introduction of many of his letters (Table 1). Saint Paul was also convinced that his sufferings and his spiritual path should be regarded as an inspiration model for Christians: *“Howbeit for this cause I obtained mercy, that in me first Jesus Christ might shew forth all longsuffering, for a pattern to them which should hereafter believe on him to life everlasting”* (1 Timothy 1:16).

### Philosophical concerns

Saint Paul’s Epistles address several theological issues and are seminal landmarks of Christian doctrines. For example, his letters address Soteriology (doctrine regarding salvation; Romans 8:28–39; Ephesians 1:3–14), Eschatology (the final events of history; 1 Thessalonians 4:13–18), and Ecclesiology (1 Corinthians 12:12–30), among others^11^. Saint Paul’s writings also dialogue with Greek philosophers^10,11^. The well-known passage on charity (1 Corinthians 13), for instance, dialogues with platonic realism and the allegory of the cave: *“For now we see through a glass, darkly; but then face to face: now I know in part; but then shall I know even as also I am known.”* (1 Corinthians 13:12).

It is also worth mentioning the debate between Saint Paul and Epicureans and Stoics philosophers regarding the origin of the world (Acts 11:18–34). While the Epicurean philosophy denied Divine intervention as the origin of the universe and supported atomic materialism, Saint Paul sided with the Stoic doctrine postulating that the world was created by Divine intervention. Moreover, his discourse against the Epicurean doctrine reflects the writings of Seneca the Younger (c. 4 BC–65 AD), the famous Roman Stoic philosopher^12^. In sum, the Epistles reflect Saint Paul’s philosophical and metaphysical concerns (Table 1).

### Hypermoralism

Besides addressing theological questions, his letters also contained behavioral recommendations for the Christian communities. Saint Paul firmly condemned sexual promiscuity, drunkenness, and homosexuality (Table 1). These recommendations were in clear contrast with the hedonism of Hellenic culture, which was dominant in Tarsus and other cities of Asia Minor during the early years of the Christian Church^10^. The following text illustrates Saint Paul’s strict moral standards, in which it is also noticeable his meticulous and sequencing writing that resembles viscosity:

*“And likewise also the men, leaving the natural use of the woman, burned in their lust one toward another; men with men working that which is unseemly, and receiving in themselves that recompense of their error which was meet. And even as they did not like to retain God in their knowledge, God gave them over to a reprobate mind, to do those things which are not convenient; Being filled with all unrighteousness, fornication, wickedness, covetousness, maliciousness; full of envy, murder, debate, deceit, malignity; whisperers, backbiters, haters of God, despiteful, proud, boasters, inventors of evil things, disobedient to parents, Without understanding, covenant breakers, without natural affection, implacable, unmerciful: Who knowing the judgment of God, that they which commit such things are worthy of death, not only do the same, but have pleasure in them that do them.”*
(Romans 1:27–31).

### Hyposexuality

In line with his hypermorality, Saint Paul recommended men not to have intimacy with women (“*It is good for a man not to touch a woman”;* 1 Corinthians 7:1). For him, the marriage was allowed by God to avoid the sin of sexual impurity: “*Nevertheless, to avoid fornication, let every man have his own wife, and let every woman have her own husband.”* (1 Corinthians 7:2).

Saint Paul was not married and practiced sexual chastity, exemplifying hyposexuality. Therefore, he recommended chastity for Christians: *“For I would that all men were even as I myself. (…). I say therefore to the unmarried and widows, it is good for them if they abide even as I. But if they cannot contain, let them marry: for it is better to marry than to burn”.* (1 Corinthians 7:7–9).

### Hypergraphy and viscosity

Saint Paul did not personally write most of his letters but dictated them to an amanuensis^13^. He probably had low vision, preventing him from writing^7^. Given that Saint Paul is the alleged author of thirteen canonical Epistles, and that other Pauline texts were possibly lost^13^, it is possible that he had a hypergraphic trace, i.e., a tendency of writing profusely, to publicize his messages through scribbled lines.

Of note, his texts are meticulous and sometimes repetitive and with marked sequencing and circumstantiality–features of viscosity (Table 1). His writings are frequently redundant, reflecting his recurrent ambition of producing all-embracing, in-depth texts. Meticulosity and sequencing can be observed in the following passage where hypermorality is also evident: *“Now the works of the flesh are manifest, which are these; adultery, fornication, uncleanness, lasciviousness, idolatry, witchcraft, hatred, variance, emulations, wrath, strife, seditions, heresies, envyings, murders, drunkenness, revellings, and such like: of the which I tell you before, as I have also told you in time past, that they which do such things shall not inherit the kingdom of God.”* (Galatians 5:19–21).

### Emotionality and irritability

Saint Paul was a man of strong convictions and highly committed to his duties. Before his conversion, he was extremely zealous regarding Jewish traditions (Galatians 1:14) and was a dreaded persecutor of Christians (Acts 8:3; Philippians 3:6; Galatians 1:13,23). After his Epiphany, Saint Paul’s firm positions and temper raised interpersonal conflicts. For instance, the Apostle had a serious quarrel with his friend Barnabas at Antioquia (Acts 15:2) and decided to travel to Syria and Cilicia without him (Acts 15:36–40). Saint Paul even disputed with the Father of the Church, Saint Peter, at Antioquia, and strongly rebuked him in his Epistle to the Galatians (2:11–14).

Moreover, the letters of Saint Paul were frequently written in a resolute and passionate style (Romans 8:31–39; 2 Corinthians 11:1–30), as follows: *“But God hath chosen the foolish things of the world to confound the wise; and God hath chosen the weak things of the world to confound the things which are mighty; and base things of the world, and things which are despised, hath God chosen, yea, and things which are not, to bring to nought things that are”* (1 Corinthians 1:27,28).

## DISCUSSION

Epileptic disorders have affected illustrious characters throughout history. The way it is reflected in historiographical texts and artistic creations has the potential to inform about a specific time in history and how that society perceived epilepsy and other neurological diseases^14^. Accordingly, it is potentially interesting and revealing the analysis of figures who suffered from epilepsy. In this manuscript, we addressed Saint Paul, more specifically, the possibility he had personality traits seen in people with TLE^5,7^. We provided an in-depth analysis of GGS features through Saint Paul’s writings and examined how his theological thinking might reflect elements of his personality traits.

At first, we must emphasize that no direct causal relationship can be established between Saint Paul’s personality traits and his theological doctrines. The assumption of complete and exclusive dependence of mental life on neural underpinnings would represent a simple form of “medical materialism”, as pointed out by William James^15^. Given the complexity of Pauline texts, cultural, historical, philosophical, and religious aspects should always be taken into account in any reassessment of Saint Paul. Conversely, the “neurotheological”^5^ approach does not necessarily exclude these factors but complements them. We propose that the study of the elements of GGS, commonly related to the psychopathology of TLE, may open a window for the interpretation of Saint Paul’s life, thinking process, and beliefs shown in his work.

Behavioral changes and religious convictions related to epilepsy have been reported for more than four thousand years^1^. In the *fin de siècle*, neuropsychiatrists described the paradoxical temper, which opposed angry outbursts to ethical and religious concerns, and the sticky-adhesive personality (viscosity) associated with seizure disorders^16^. In the middle of the 20th century, the concept of GGS gained its distinctive key points. Henri Gastaut described that TLE-associated features such as emotional intensity, hyposexuality, and stickiness of attention (viscosity) contrasted with Klüver-Bucy syndrome, in which patients with bilateral temporal damage exhibit placidity, hypersexuality, and flight attention^17^. These observations reinforced the role of temporal lobes in mood, cognition, and behavior. In the 1970s, Norman Geschwind’s studies validated the early findings linking deepened emotions to temporal lobes, also including hypergraphic, philosophical interests, and religiosity traits in the spectrum of TLE-related interictal behaviors^18^. In the 1990s, Blumer et al.^16^ proposed objective measures of GGS by adapting the Bear-Fedio Inventory (BFI)^19^ into the Neurobehavior Inventory (NBI)^16^. The NBI measures twenty items, including anger and temper, suspicion, interest in details, writing tendency, sense of personal destiny, sense of law and order, cosmic interests, feelings about sex, and other features related to the GGS^16^. More recently, neuroimaging studies demonstrated the association of religiosity and sense of personal destiny with right temporal lobe pathology in patients with refractory TLE^3,20^.

Hyperreligiosity is a core feature of the GGS and is indissociably linked to Saint Paul. For him, the religious experience could be referred to as *“an acute fever,”* as classically described by William James (Table 2). Man of strong convictions and powerful rhetoric, Saint Paul communicated his ideas in an implacable and emotional style. For instance, he presented an impressive and meticulous picture of the human’s sinful condition in the first chapters of the Epistle to the Romans, highlighting the inescapable culpability of humankind. Here again, his theological view is embedded with moral concerns, emotionality, and viscosity — elements of the GGS.

**Table 2:** Excerpt from “Lecture I: Religion and Neurology”, from “The Varieties of Religious Experience”, by William James (1902).

*“There can be no doubt that as a matter of fact a religious life, exclusively pursued, does tend to make the person exceptional and eccentric. I speak not now of your ordinary religious believer, who follows the conventional observances of his country, whether it be Buddhist, Christian, or Mohammedan. His religion has been made for him by others, communicated to him by tradition, determined to fixed forms by imitation, and retained by habit. It would profit us little to study this second-hand religious life. We must make search rather for the original experiences which were the pattern-setters to all this mass of suggested feeling and imitated conduct. These experiences we can only find in individuals for whom religion exists not as a dull habit, but as an acute fever rather. But such individuals are “geniuses” in the religious line; and like many other geniuses who have brought forth fruits effective enough for commemoration in the pages of biography, such religious geniuses have often shown symptoms of nervous instability. Even more perhaps than other kinds of genius, religious leaders have been subject to abnormal psychical visitations. Invariably they have been creatures of exalted emotional sensibility. Often, they have led a discordant inner life, and had melancholy during a part of their career. They have known no measure, been liable to obsessions and fixed ideas; and frequently they have fallen into trances, heard voices, seen visions, and presented all sorts of peculiarities which are ordinarily classed as pathological. Often, moreover, these pathological features in their career have helped to give them their religious authority and influence.”*

The sense of destiny, a feature of the GGS, is markedly present throughout Saint Paul’s Epistles and aligns with the doctrine of Election and Predestination. According to this doctrine, humankind is unable to choose Christ due to its original sinful condition. Therefore, Salvation is a sovereign act of God, Who elects (determines) those who will become part of His people^11^. Therefore, the Grace of God makes Salvation possible. The Arminian theology refuses the doctrine of Election and Predestination which is a main principle of Calvinism (*Sola Gratia*). Interestingly, several Pauline texts (Romans 8:28–39; Ephesians 1:3–14; 2 Thessalonians 2:13,14; 2 Timothy 1:9,10) support the Predestination and Election doctrines^11^, raising the question of whether Saint Paul’s sense of destiny influenced his view about Salvation. The firm belief of Saint Paul in his Revelation and Vocation (Romans 1:1; 1 Corinthians 1:1; 2 Corinthians 1:1; Galatians 1:1; 11,12; 1 Timothy 1:16; 2 Timothy 1:1) also exerted an influence on his conception about the Divine inspiration of his writings (2 Timothy 3:16,17), which also became a founding principle of Christianism, especially in Calvinism (*Sola Scriptura*).

We are aware that several arguments may be raised against our perspective. First, Saint Paul’s diagnosis of epilepsy is not consensual among historians^21,22^ and there are authors with strong arguments endorsing a mood disorder with psychotic features^23^. However, there are supporting elements that his “*thorn in the flesh*” was an epileptic disorder, with ecstatic auras^5,7,8^. Importantly, the presence of elements of GGS *per se* corroborates the hypotheses that Saint Paul had epilepsy. Second, the source of our investigation was the New Testament’s Epistles attributed to Saint Paul. There is a debate about the authorship of some of his letters^13^. While some Epistles (Romans, 1 Corinthians, 2 Corinthians, Galatians, Philippians, 1 Thessalonians, and Philemon) were undoubtedly written by Saint Paul, others (Ephesians, Colossians, 2 Thessalonians, 1 and 2 Timothy, and Titus) were probably written by his disciples and are considered as Deutero-Paulines^13^. To minimize that, in the current study, we analyzed the New Testament’s Epistles attributed to him following Christian tradition. Third, it should be recognized that hyperreligiosity and sense of destiny are common personality features of Christian Saints and are observed not only in epileptic people. Finally, epilepsy and the related GGS personality traits do not minimize the spiritual and theological contributions and/or values of Saint Paul’s texts^8^, as Dostoevsky’s epilepsy does not devalue his literature^24^. Indeed, Fyodor Dostoevsky (1821–1881) suffered from epilepsy during most of his life. While epilepsy severely impacted his quality of life, the disease did not depreciate his literary work. On the contrary, some of his novels (e.g., “The Idiot”) offer a valuable window to understand the representation of epilepsy in Western culture, and provide an extraordinary example of the interface between genius and neurological diseases.

In sum, behavioral aspects suggestive of GGS shed light on the personality and theological production of Saint Paul, a genius man of intellectual excellence and refined culture, whose life and writings exerted a decisive influence on Western history.
